# Pharmacological properties of JTE-052: a novel potent JAK inhibitor that suppresses various inflammatory responses in vitro and in vivo

**DOI:** 10.1007/s00011-014-0782-9

**Published:** 2014-11-12

**Authors:** Atsuo Tanimoto, Yoshihiro Ogawa, Chika Oki, Yukari Kimoto, Keisuke Nozawa, Wataru Amano, Satoru Noji, Makoto Shiozaki, Akira Matsuo, Yuichi Shinozaki, Mutsuyoshi Matsushita

**Affiliations:** Central Pharmaceutical Research Institute, Japan Tobacco Inc., 1-1 Murasaki-cho, Takatsuki, Osaka 569-1125 Japan

**Keywords:** JTE-052, Janus kinase, Cytokine signaling, Collagen-induced arthritis

## Abstract

**Objective:**

To evaluate the pharmacological properties of JTE-052, a novel Janus kinase (JAK) inhibitor.

**Methods:**

The JAK inhibitory activity of JTE-052 was evaluated using recombinant human enzymes. The inhibitory effects on cytokine signaling pathways were evaluated using primary human inflammatory cells. The in vivo efficacy and potency of JTE-052 were examined in a mouse interleukin (IL)-2-induced interferon (IFN)-γ production model and a rat collagen-induced arthritis model.

**Results:**

JTE-052 inhibited the JAK1, JAK2, JAK3, and tyrosine kinase (Tyk)2 enzymes in an adenosine triphosphate (ATP)-competitive manner and inhibited cytokine signaling evoked by IL-2, IL-6, IL-23, granulocyte/macrophage colony-stimulating factor, and IFN-α. JTE-052 inhibited the activation of inflammatory cells, such as T cells, B cells, monocytes, and mast cells, in vitro. Oral dosing of JTE-052 resulted in potent suppression of the IL-2-induced IFN-γ production in mice with an ED_50_ value of 0.24 mg/kg, which was more potent than that of tofacitinib (ED_50_ = 1.1 mg/kg). In the collagen-induced arthritis model, JTE-052 ameliorated articular inflammation and joint destruction even in therapeutic treatments where methotrexate was ineffective.

**Conclusions:**

The present results indicate that JTE-052 is a highly potent JAK inhibitor, and represents a candidate anti-inflammatory agent for suppressing various types of inflammation.

**Electronic supplementary material:**

The online version of this article (doi:10.1007/s00011-014-0782-9) contains supplementary material, which is available to authorized users.

## Introduction

The family of cytokines that bind type I and type II cytokine receptors, including interleukins (ILs), interferons (IFNs), and colony-stimulating factors, as well as classic hormones such as erythropoietin, prolactin, and growth hormone [1], are important for acquired and innate immunity and hematopoiesis. Signaling via these receptors is dependent on a small family of structurally distinct kinases named the Janus kinases (JAKs). The JAK family contains four members, namely JAK1, JAK2, JAK3, and Tyk2 [2], which selectively associate with the membrane proximal domains of type I and II cytokine receptors through various combinations of JAKs. Upon ligand binding, JAKs phosphorylate the cytokine receptors and induce the recruitment of various signaling proteins, including the signal transducers and activators of transcription (Stat) family, which directly modulate gene expression as transcription factors.

Several small-molecule JAK inhibitors are currently under clinical development [3]. Tofacitinib represents the first small molecule developed as a selective inhibitor of the JAKs, and exhibits nanomolar potency and a high degree of kinome selectivity [4]. In clinical trials on patients with rheumatoid arthritis (RA), it was demonstrated that tofacitinib was highly efficacious even in patients with inadequate responses to conventional disease-modifying antirheumatic drugs (DMARDs) such as methotrexate (MTX) [5, 6]. It was also reported that tofacitinib showed efficacy in various inflammation-related diseases such as inflammatory bowel disease (IBD) [7], psoriasis [8], and transplant rejection [9]. Tofacitinib is a selective inhibitor for the JAK family of kinases and has been reported to block the cytokine signaling related to JAK1/3 [10, 11] and inhibit the function of T cells [12], but the precise mechanism by which tofacitinib shows such broad efficacy is not well understood. There are several reports regarding adverse events, including elevation of transaminases or rates of infection, in tofacitinib-treated patients [5, 6]. The elevation of transaminases is considered to be an off-target effect, whereas the elevation of infection, the rates of which were no greater than those of biologics [13], is considered to be an on-target effect. These adverse events may limit the usage of tofacitinib in clinical settings, suggesting that new JAK inhibitors with distinct profiles from that of tofacitinib are needed to conclude the value of the JAK family kinases as therapeutic targets for inflammation-related diseases.

We have identified a novel and specific JAK inhibitor, JTE-052, through a medical chemistry campaign. In the present study, we characterized the in vitro and in vivo pharmacological profiles of JTE-052. In our in vitro experiments, we investigated the inhibitory effects on JAK enzymes and cytokine signaling pathways, and compared these effects with other known JAK inhibitors. In addition, the effects of JTE-052 on the activation of various types of inflammatory cells were investigated. In our in vivo experiments, the inhibitory effects of JTE-052 on cytokine signaling were investigated, and the potency was compared with that of tofacitinib. In addition, the antirheumatic effects of JTE-052 were investigated in rats with collagen-induced arthritis under the MTX-resistant condition.

## Materials and methods

### Animals

All animals were obtained from Charles River Laboratories Japan (Yokohama, Japan) and maintained under specific pathogen-free conditions at a room temperature of 23 ± 3 °C and air humidity of 55 ± 15 % on a 12-h/12-h light/dark cycle. This animal study was conducted in accordance with the Japanese Law for the Humane Treatment and Management of Animals (Law No. 105, October 1, 1973). Prior to the initiation of the animal study, the outline animal study protocol had been reviewed by the Institutional Animal Care and Use Committee of the Biological/Pharmacological Research Laboratories, Central Pharmaceutical Research Institute, Japan Tobacco Inc.

### Compounds and reagents

JTE-052 and tofacitinib citrate were prepared in a manner described elsewhere (WO 2011013785 A1 or US 20030130292 A1) at the Central Pharmaceutical Research Institute, Japan Tobacco Inc. (Osaka, Japan). Ruxolitinib phosphate was purchased from Haoyuan Chemexpress Co., Ltd. (Shanghai, P. R. China). Methotrexate hydrate (MTX) was purchased from Sigma-Aldrich, Co. (St. Louis, MO). For the in vitro experiments, JTE-052 was dissolved in dimethyl sulfoxide (DMSO) and diluted with the buffer used in each experiment. For the in vivo experiments, JTE-052 and MTX were suspended in 0.5 % (w/v) methyl cellulose solution.

Recombinant human IL-2, IL-6, IL-21, and granulocyte/macrophage colony-stimulating factor (GM-CSF), and recombinant mouse IL-2 were purchased from PeproTech Inc. (Rocky Hill, NJ). Recombinant human IL-4 was from R&D Systems Inc. (Minneapolis, MN). Recombinant human IFN-α was from Miltenyi Biotec Inc. (Bergisch Gladbach, Germany). Recombinant human IL-23 was from HumanZyme Inc. (Chicago, IL).

PE-conjugated antibodies against pStat1, pStat3, and pStat5, PE-Cy7-conjugated antibody against CD3, and FITC-conjugated antibody against CD4 were purchased from BD Biosciences (San Jose, CA).

Recombinant kinase domains of human JAK1 (850–end) and Tyk2 (871–end) were purchased from Carna Biosciences Inc. (Kobe, Japan), and those of JAK2 (808–end) and JAK3 (781–end) were from Millipore Corporation (Billerica, MA).

### Enzyme assay

Kinase assays using a recombinant enzyme, biotinylated substrate, and [^33^P]ATP were performed as previously described [14] with some modifications. Each enzyme reaction was performed with a recombinant JAK1, JAK2, JAK3, and Tyk2 enzyme (4, 0.2, 0.2, and 2 μg/mL protein, respectively), 10 μM TK substrate-biotin (Cisbio Bioassays, Codolet, France), ATP at the Km specific for each enzyme (40, 10, 2.5, and 40 μM, respectively), 50 mM HEPES (pH 7.0), 0.01 % bovine serum albumin (BSA), 0.02 % NaN_3_, 0.1 mM orthovanadate, 5 mM MgCl_2_, 1 mM dithiothreitol, 50 nM Supplement Enzymatic Buffer (Cisbio Bioassays), and 1 % DMSO.

### Primary human cells

Human peripheral blood was obtained from healthy volunteers with informed consent on the basis of the Declaration of Helsinki. Human peripheral blood mononuclear cells (hPBMCs) were purified by density centrifugation using Lymphoprep™ (Axis-Shield, Dundee, UK). Human T cells, B cells, and monocytes were isolated from hPBMCs with a Pan T Cell Isolation Kit II Human, B Cell Isolation Kit II Human (Miltenyi Biotec Inc.), and Dynabeads^®^ Untouched™ Human Monocytes (Life Technologies, Carlsbad, CA), respectively, according to the corresponding manufacturer’s instructions, and >95 % purity of the isolated cells was confirmed by flow cytometry. Human mast cells were obtained from CD34^+^ cord blood cells (Lonza Walkersville Inc., Walkersville, MD) by culture in the presence of stem cell factor (SCF), IL-6, and IL-3 for 10 days, followed by culture in the presence of SCF and IL-6 for 8 weeks. Normal human lung fibroblasts (NHLFs) were purchased from Lonza Walkersville Inc.

### Flow cytometry analysis

hPBMCs (3 × 10^6^ cells/tube) were incubated with a test compound for 30 min at 37 °C, and then treated with IL-2 (100 ng/mL), IL-6 (100 ng/mL), IL-23 (100 ng/mL), IFN-α (100 ng/mL), or GM-CSF (1 ng/mL) for an additional 15 min. To terminate the stimulation, the cells were fixed with Fixation Buffer (BD Biosciences) for 10 min at 37 °C. The fixed cells were incubated with Perm Buffer II (BD Biosciences) for 30 min on ice and then incubated with fluorochrome-labeled anti-CD3, anti-CD4, or anti-phospho-Stat antibodies for 30 min at room temperature. The cytokine-induced Stat phosphorylation was analyzed and quantified using a Cytomics FC500 (Beckman Coulter Inc., Brea, CA). In the case of IL-23 stimulation, hPBMCs were precultured with 10 μg/mL phytohemagglutinin (PHA)-M (Sigma-Aldrich Co.) for 3 days to enhance their response to IL-23.

### Cellular assays

For determination of IL-2-induced T cell proliferation, human T cells were precultured with 10 μg/mL PHA-M for 3 days and plated in 96-well plates at 1.0 × 10^4^ cells/well in the presence or absence of various concentrations of JTE-052. Following preincubation with the compound for 30 min at 37 °C, the cells were stimulated by adding 20 ng/mL recombinant human IL-2 to each well and incubated for 3 days at 37 °C under 5 % CO_2_. [^3^H]Thymidine (37 kBq) was added during the culture period. After completion of the culture period, the cells were harvested with a 96-well harvester and counted in a scintillation counter.

For determination of IL-21-induced B cell proliferation, human B cells were plated in 96-well plates at 1 × 10^5^ cells/well in the presence or absence of JTE-052. Following preincubation with the compound for 30 min, the cells were stimulated by adding 10 ng/mL recombinant human IL-21 and 1 μg/mL anti-CD40 antibody. The cells were then cultured for 3 days, and [^3^H]thymidine uptake was measured as described above.

For determination of GM-CSF-induced tumor necrosis factor (TNF)-α production from monocytes, human monocytes were plated in 96-well plates at 1 × 10^5^ cells/well in the presence or absence of JTE-052. Following preincubation with the compound for 30 min, the cells were stimulated by adding 0.03 ng/mL recombinant human GM-CSF for 16 h. The cells were then cultured with 0.1 ng/mL lipopolysaccharide (LPS) for an additional 6 h at 37 °C. The supernatants were collected, and secretion of TNF-α was measured by ELISA.

For determination of IL-4-induced TNF-α production from mast cells, human mast cells were plated in 96-well plates at 2 × 10^5^ cells/well in the presence or absence of JTE-052. Following preincubation with the compound for 10 min, the cells were stimulated by adding 10 ng/mL recombinant human IL-4 and 1 μg/mL immunoglobulin (Ig)E for 5 days. The cells were then replated at 1.2 × 10^5^ cells/well and cultured with 1 μg/mL anti-IgE for an additional 5 h at 37 °C. The supernatants were collected, and secretion of TNF-α was measured by ELISA.

For determination of cytotoxicity, fibroblast proliferation was measured. NHLFs were plated in 96-well plates at 1 × 10^3^ cells/well in the presence or absence of JTE-052 without the addition of cytokines. The cells were then cultured for 3 days, and [^3^H]thymidine uptake was measured as described above.

The cells were cultured at 37 °C under 5 % CO_2_ in RPMI1640 medium containing 10 % fetal calf serum (T cells, B cells, monocytes, and NHLFs) or Iscove’s modified Dulbecco’s medium containing 0.1 % BSA, 55 µM 2-mercaptoethanol, and 1 % Insulin-Transferrin-Selenium, 100x (Life Technologies) (Mast cells).

### IL-2-induced IFN-γ production in mice

DBA/1J mice were dosed once via oral gavage with compounds. At 1 or 6 h after compound administration, the mice were injected intraperitoneally with 2 μg of recombinant mouse IL-2 and 10 μg of biotinylated anti-mouse IFN-γ capture antibody. After 6 h, the mice were euthanized, and blood was collected. Serum IFN-γ was quantified using a Mouse IFN-γ In Vivo Capture Assay Set (BD Biosciences).

### Collagen-induced arthritis in rats

CIA was induced in rats as previously described [15] with some modifications. Briefly, type II collagen (Collagen Research Center, Saitama, Japan) was dissolved in 0.01 M acetic acid at 2 mg/mL, and the solution was emulsified in an equal volume of Freund’s incomplete adjuvant (Difco Laboratories, Detroit, MI). Lewis rats were immunized with 1 mL of the emulsion (1 mg of type II collagen) via ten intradermal injections on the back under anesthesia. The rats were then challenged with 0.2 mL of the emulsion injected into the base of the tail on day 8 under anesthesia. The test drugs were given orally once daily from day 1 to day 21 (preventive administration) or from day 15 to day 28 (therapeutic administration). After arthritis induction, the hind paw volume was measured by a water displacement method using a plethysmometer (Muromachi Kikai Co. Ltd., Tokyo, Japan). On day 22 or day 29, the rats were euthanized, and their hind paws were excised and X-rayed or processed for histological evaluation. Radiographs of the right hindlimbs were taken using a microfocal cone-beam X-ray CT scanner (MCT-CB100MF; Hitachi Medical Corporation, Tokyo, Japan). The severity of bone destruction was scored on a 3-point scale ranging from 0 to 2 for the following five joints: second metatarsal—second intertarsal joint, third metatarsal—third intertarsal joint, talus—central intertarsal joint, calcaneus—fourth intertarsal joint, and astragalo—tibial joint. For histological analysis, the formalin-fixed left hindlimbs were sectioned and stained with hematoxylin and eosin. The histology of the tarsal joints was evaluated by assessing the following parameters defined in the preliminary examination: inflammatory cell infiltration, synovial cell hyperplasia, cartilage destruction, and bone destruction. The severity of each histological change was scored on a 5-point scale ranging from 0 to 4. (Score 0: normal; Score 1: minimal, solitary (and very small) lesion; Score 2: slight, focal (and small) lesion; Score 3: moderate, scattered lesion; and Score 4: severe, extensive lesion).

### Statistical analysis

Data are expressed as the mean ± standard error of the mean (SEM) (for in vitro experiments) or mean ± standard deviation (SD) (for in vivo experiments) of the indicated numbers of samples. The statistical significance was assessed by Dunnett’s test (for homoscedastic data) or the Steel test (for heteroscedastic data) after homoscedasticity analysis by Bartlett’s test. For radiographic and histological scores, the statistical significance was assessed by the Steel test. For comparisons of the inhibitory activity of compounds in enzyme assays, the pKi values were statistically analyzed.

## Results

### JTE-052 selectively inhibits JAK activity in an ATP-competitive manner

**Fig. 1:**
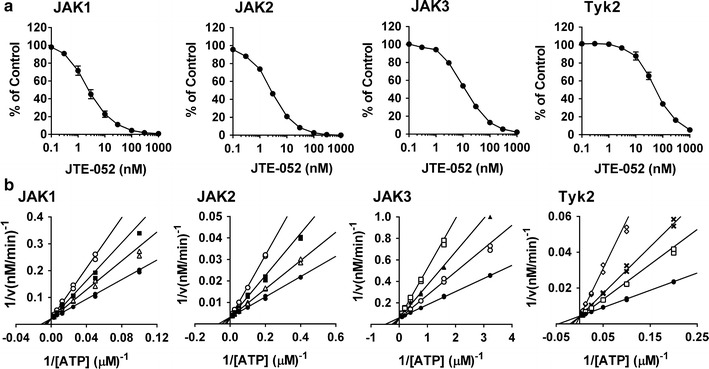
Inhibitory effects of JTE-052 on JAK enzymes. Enzyme assays were performed using the kinase domains of recombinant human JAK enzymes, a biotinylated peptide substrate, and [^33^P]ATP. **a** Inhibitory effects were determined at an ATP concentration equivalent to the apparent Km for each enzyme. **b** Lineweaver–Burk linear transformations were plotted for 0 (*filled circles*), 1 (*open triangles*), 2 (*filled squares*), 4 (*open circles*), 8 (*filled triangles*), 16 (*open squares*), 32 (*crosses*), and 64 nM (*open diamonds*) JTE-052. Three independent experiments were performed. Values are mean ± SEM (**a**). Representative data are shown from three independent experiments (**b**)

In the enzymatic assays, JTE-052 potently inhibited all of the JAK subtypes with IC_50_ values of 2.8 ± 0.6, 2.6 ± 0.2, 13 ± 0, and 58 ± 9 nM for JAK1, JAK2, JAK3, and Tyk2, respectively (Fig. 1a; Table 1). Lineweaver–Burk plots showed that the inhibition mode of JTE-052 toward all JAKs was competitive with ATP (Fig. 1b; Table 1) with Ki values of 2.1 ± 0.3, 1.7 ± 0.0, 5.5 ± 0.3, and 14 ± 1 nM for JAK1, JAK2, JAK3, and Tyk2, respectively. The selectivity of JTE-052 for other kinases was assessed using KinaseProfiler™ (Millipore Co. Ltd.). JTE-052 did not inhibit any of the kinases even at the concentration of 1,000 nM, except for ROCK-II (Table 2). Tofacitinib and ruxolitinib also inhibited all of the JAK enzymes, but the JAK3 inhibition by tofacitinib and JAK2 and Tyk2 inhibition by ruxolitinib were significantly more potent than those of JTE-052 (Tables 1, S1). Tofacitinib did not inhibit any of the kinases in Kinase Profiler™, even at a concentration of 1,000 nM.

**Table 1:** IC_50_ and Ki values of JAK inhibitors in enzyme assays

Compounds		JAK1	JAK2	JAK3	Tyk2
JTE-052	IC_50_ (nM)	2.8 ± 0.6	2.6 ± 0.2	13 ± 0	58 ± 9
	Ki (nM)	2.1 ± 0.3	1.7 ± 0.0	5.5 ± 0.3	14 ± 1
Tofacitinib	Ki (nM)	2.0 ± 0.4	1.3 ± 0.0	0.74 ± 0.01	20 ± 2
Ruxolitinib	Ki (nM)	1.3 ± 0.3	0.36 ± 0.10	7.0 ± 1.9	1.6 ± 0.1

Values are mean ± SEM. Three independent experiments were performed

**Table 2:** Kinase selectivities of JTE-052 determined by KinaseProfiler™

Kinase	IC_50_ (nM)	Kinase	IC_50_ (nM)
Abl	>1,000	Lyn	>1,000
Aurora-A	>1,000	MAPK2	>1,000
CDK1/cyclinB	>1,000	MAPKAP-K2	>1,000
CDK2/cyclinA	>1,000	MEK1	>1,000
CHK1	>1,000	Met	>1,000
CK2	>1,000	MSK1	>1,000
cKit	>1,000	p70S6 K	>1,000
CSK	>1,000	PDGFRα	>1,000
c-RAF	>1,000	PDGFRβ	>1,000
cSRC	>1,000	PDK1	>1,000
EGFR	>1,000	PKA	>1,000
FGFR1	>1,000	PKBα	>1,000
FGFR3	>1,000	PKCα	>1,000
Flt1	>1,000	PKCβI	>1,000
Flt3	>1,000	PKCγ	>1,000
Fyn	>1,000	PKCζ	>1,000
GSK3β	>1,000	Plk1	>1,000
IGF-1R	>1,000	ROCK-II	141
IKKβ	>1,000	SAPK2a	>1,000
IR	>1,000	SAPK2b	>1,000
JNK1α1	>1,000	SAPK3	>1,000
JNK2α2	>1,000	SGK	>1,000
JNK3	>1,000	Syk	>1,000
KDR	>1,000	Tie2	>1,000
Lck	>1,000	ZAP-70	>1,000

### JTE-052 inhibits various cytokine signaling pathways

**Fig. 2:**
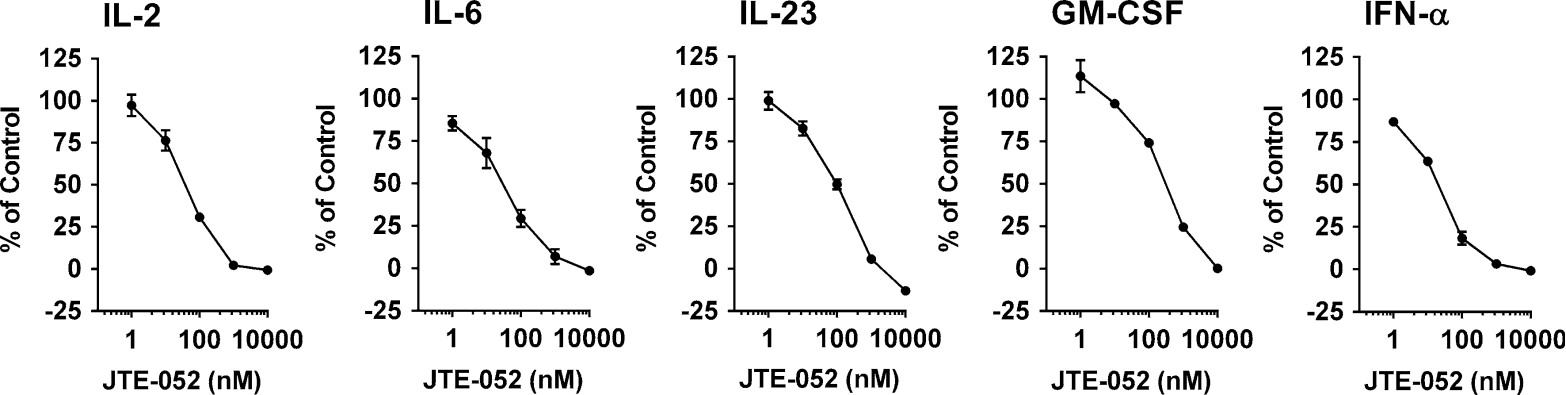
Inhibitory effects of JTE-052 on cytokine signaling. hPBMCs were stimulated with IL-2 (100 ng/mL), IL-6 (100 ng/mL), IL-23 (100 ng/mL), GM-CSF (1 ng/mL), or IFN-α (100 ng/mL) for 15 min in the presence or absence of JTE-052. The phosphorylation levels of Stat5 (IL-2 and GM-CSF), Stat3 (IL-6 and IL-23), or Stat1 (IFN-α) in CD3^+^CD4^+^ cells (IL-2, IL-23, and IFN-α) or CD3^-^CD4^+^ cells (IL-6 and GM-CSF) were analyzed by flow cytometry. Data are presented as percentages of mean fluorescence intensity relative to vehicle (100 %) and non-stimulated control cells (0 %). Values are mean ± SEM. Three independent experiments were performed

To assess the inhibitory activities of JTE-052 on cytokine signaling pathways, the phosphorylation of Stat proteins was measured in hPBMCs upon cytokine stimulation. In these cell-based cytokine signaling assays, JTE-052 inhibited the phosphorylation of Stat proteins induced by IL-2, IL-6, IL-23, GM-CSF, and IFN-α with IC_50_ values of 40 ± 9, 33 ± 14, 84 ± 11, 304 ± 22, and 18 ± 3 nM, respectively (Fig. 2; Table 3). Tofacitinib and ruxolitinib also inhibited these cytokine signaling pathways. The inhibition profile of tofacitinib for the cytokine signaling pathways was almost same as that of JTE-052, while ruxolitinib was more potent than JTE-052, especially for the signaling induced by IL-23, GM-CSF, and IFN-α (Table 3).

**Table 3:** IC_50_ values in cytokine signaling

Compounds	IL-2	IL-6	IL-23	GM-CSF	IFN-α
	JAK1/JAK3	JAK1/JAK2	JAK2/Tyk2	JAK2/JAK2	JAK1/Tyk2
JTE-052 IC_50_ (nM)	40 ± 9	33 ± 14	84 ± 11	304 ± 22	18 ± 3
Tofacitinib IC_50_ (nM)	15	40	254	164	15
Ruxolitinib IC_50_ (nM)	13	19	3.6	48	1.1

Values are mean ± SEM. Three independent experiments were performed for JTE-052, and single experiment was performed for tofacitinib and ruxolitinib

### JTE-052 inhibits the activation of inflammatory cells in vitro

**Fig. 3:**
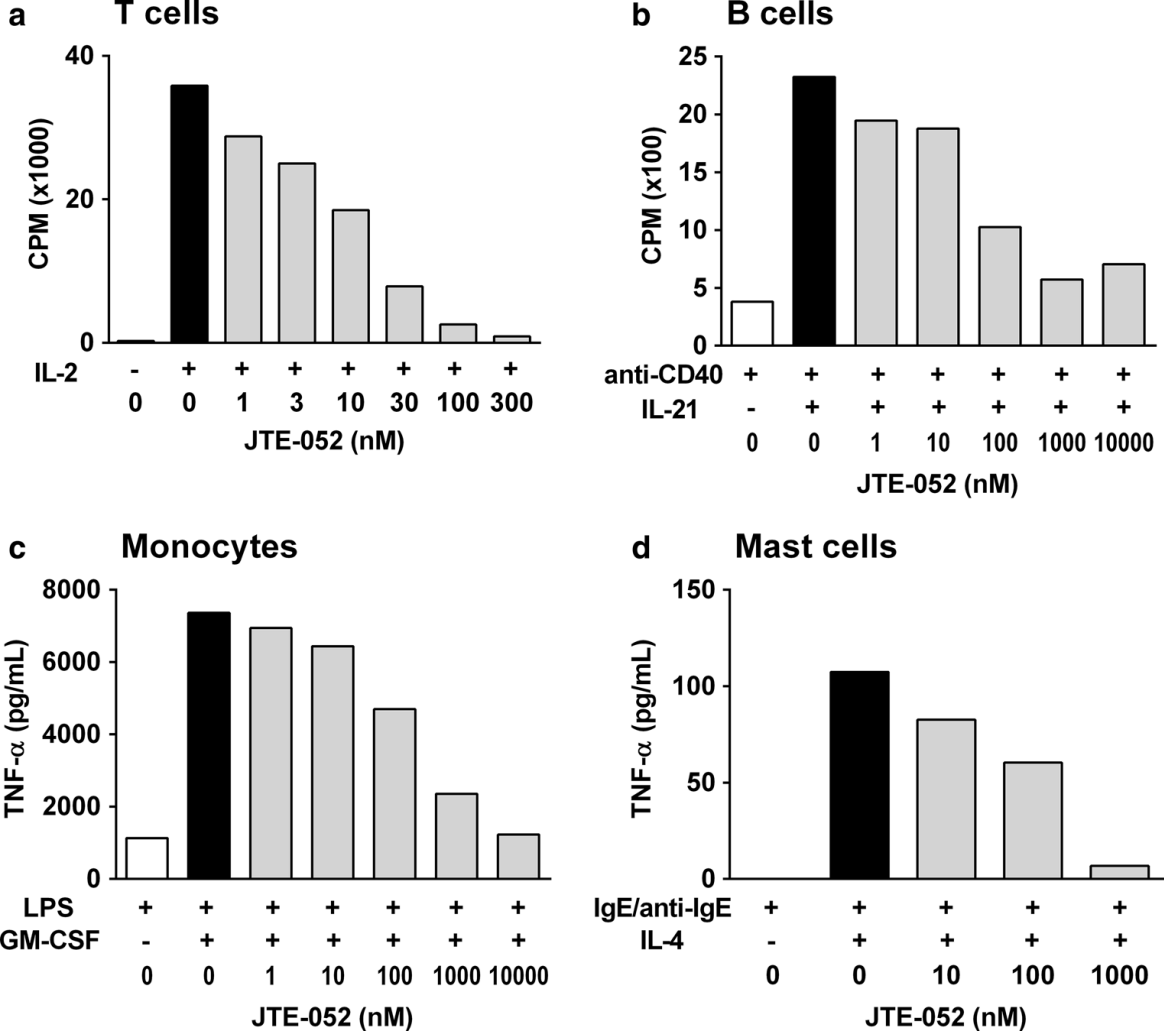
Inhibitory effects of JTE-052 on the activation of various inflammatory cells. **a** Human T cells pretreated with PHA were stimulated with IL-2 (20 ng/mL) for 3 days in the presence of JTE-052. **b** Human B cells were stimulated with IL-21 (10 ng/mL) and anti-CD40 antibody (1 μg/mL) for 3 days in the presence of JTE-052. Proliferation was estimated using [^3^H]thymidine. **c** Human monocytes were stimulated with GM-CSF (0.03 ng/mL) for 16 h in the presence of JTE-052, and then stimulated with LPS (0.1 ng/mL) for an additional 6 h. **d** Human mast cells were stimulated with IL-4 (10 ng/mL) and IgE (1 μg/mL) for 5 days in the presence of JTE-052, and simulated with anti-IgE antibody (1 μg/mL) for an additional 5 h. The amounts of TNF-α in the supernatants were measured by ELISA. Three independent experiments using cells from different donors were performed. Values are the means of 3–6 samples from one representative experiment

Next, the effects of JTE-052 on the activation of inflammatory cells were investigated. Other JAK inhibitors, such as tofacitinib, were reported to inhibit T cell proliferation [12]. Therefore, the inhibitory effect of JTE-052 to the proliferation of T cells was initially determined. JTE-052 inhibited IL-2-induced proliferation of T cells in a concentration-dependent manner (Figs. 3a, S1a; IC_50_ = 8.9 ± 3.6 nM), and its potency was similar to that of tofacitinib (IC_50_ = 16 nM). We also evaluated the effect of JTE-052 on normal cell proliferation using NHLFs and found that JTE-052 did not show clear inhibitory effect on NHLF proliferation (IC_50_ > 10,000 nM). These results indicated that the inhibitory effect of JTE-052 on the proliferation of T cells was not caused by cytotoxicity.

Next, we examined the effects of JTE-052 on B cells, monocytes, and mast cells, because cytokines play an important role in the activation of these cell types. B cells are considered to play a role in the pathogenesis of autoimmune diseases and allergic diseases through the production of autoreactive or antigen-specific antibodies. IL-21 plays an important role in B cell proliferation at the germinal center during the course of antigen-specific B cell development [16]. As shown in Figs. 3b and S1b, human B cells underwent proliferation upon stimulation with IL-21 together with anti-CD40 antibody, and JTE-052 inhibited this proliferation in a concentration-dependent manner (IC_50_ = 49 ± 6 nM).

Monocytes and macrophages are activated by various kinds of mediators, such as GM-CSF, and play important roles in inflammatory diseases such as RA [17]. In human monocytes, GM-CSF enhanced the TNF-α production upon LPS stimulation, and JTE-052 inhibited this enhancement of TNF-α production in a concentration-dependent manner (Figs. 3c, S1c; IC_50_ = 277 ± 146 nM).

Mast cells are reported to play an important role in the pathogenesis of allergic diseases such as asthma. It has been reported that IL-4 is an activator of mast cells and induces the expression of Fcε receptors on their cell surface [18]. In mast cells, TNF-α production induced by IgE receptor crosslinking was primed by IL-4, and JTE-052 inhibited this priming effect of IL-4 on Fcε receptor-dependent TNF-α production in a concentration-dependent manner (Figs. 3d, S1d; IC_50_ = 133 ± 18 nM).

### JTE-052 potently inhibits the inflammatory response in vivo

**Fig. 4:**
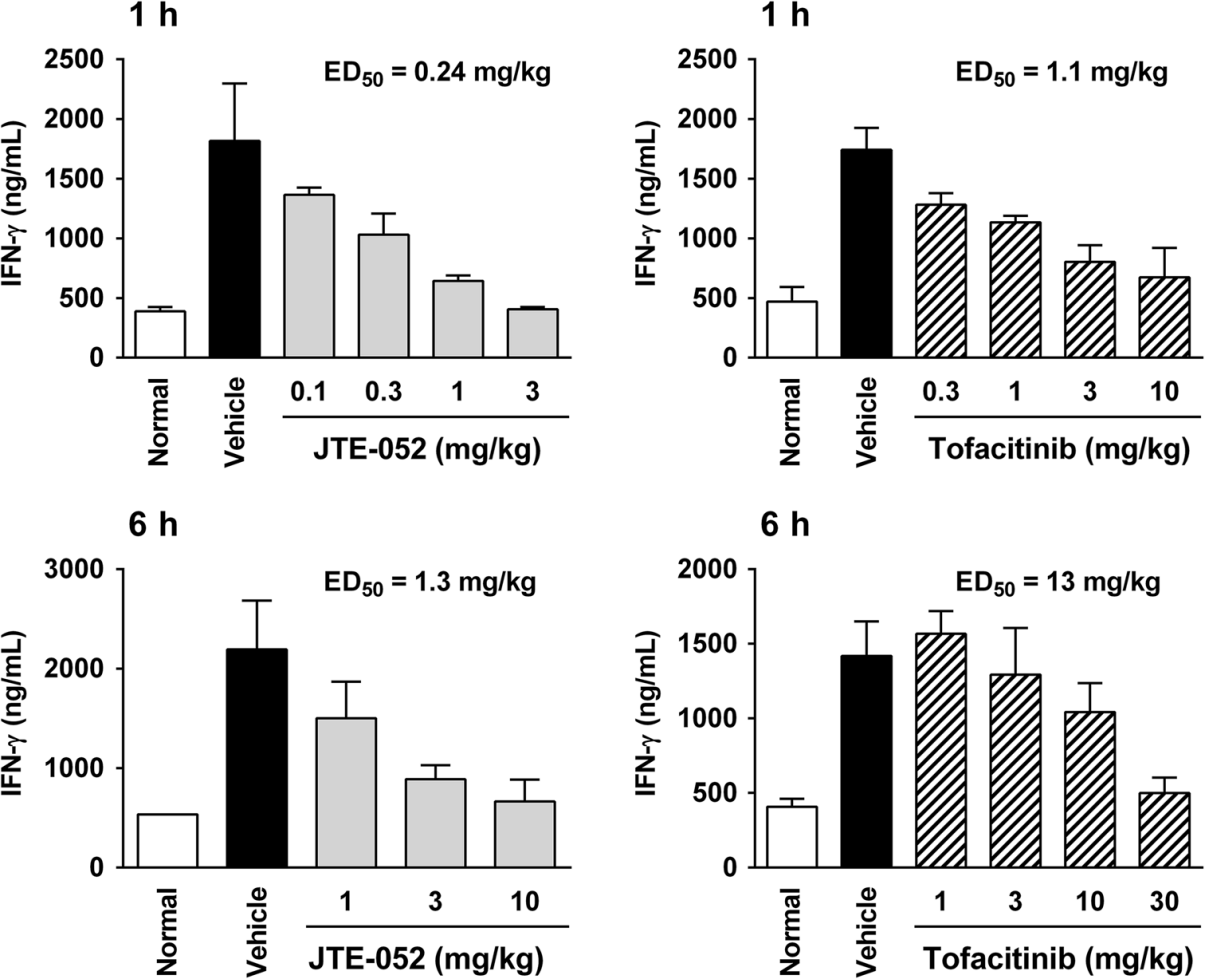
In vivo potency of JTE-052. DBA/1J mice (*n* = 5–10) were dosed with vehicle, JTE-052, or tofacitinib. After 1 or 6 h, they were injected with IL-2 and anti-mouse IFN-γ capture antibody. At 6 h after the injection, blood samples were collected, and the serum IFN-γ levels were measured. Values are mean ± SD

Next, we examined the anti-inflammatory effects of JTE-052 in vivo. Mice were intraperitoneally injected with recombinant mouse IL-2, and their IFN-γ production levels in plasma were determined. JTE-052 was orally administered at 1 or 6 h prior to the IL-2 injection. In both cases, JTE-052 decreased the IFN-γ production, but the potency of the 1-h prior administration was higher than that of the 6-h prior administration (Fig. 4; ED_50_ = 0.24 versus 1.3 mg/kg).

Tofacitinib had an inhibitory effect in the mouse model (Fig. 4), but its potency (ED_50_ = 1.1 and 13 mg/kg at 1 and 6 h, respectively) was more than 5–10 times lower than that of JTE-052.

### JTE-052 ameliorates collagen-induced arthritis in rats

**Fig. 5:**
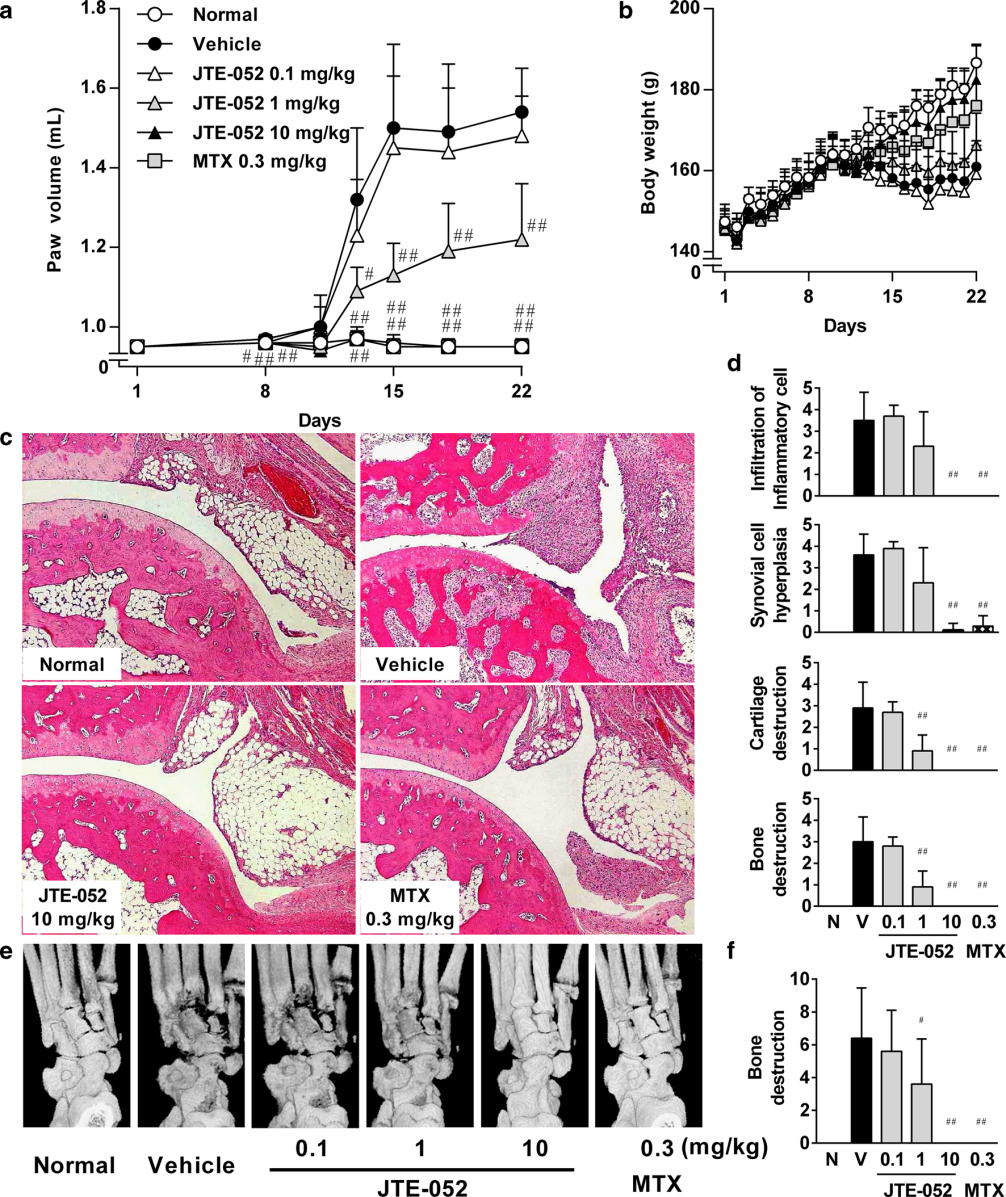
Preventive effects of JTE-052 on collagen-induced arthritis in a rat model. Rats (*n* = 10) were orally administered vehicle, JTE-052 (0.1, 1, or 10 mg/kg), or MTX (0.3 mg/kg) once daily for 3 weeks from the day of the first collagen injection. The hind paw volume (**a**) and body weight (**b**) were measured periodically. Images of hematoxylin and eosin staining (**c**) and radiographs of hindlimbs (**e**) are shown. Histological change (**d**) and radiographical change (**f**) were scored. The results are expressed as mean ± SD. ^#^ *p* < 0.05, ^##^ *p* < 0.01, versus vehicle by the Steel test

**Fig. 6:**
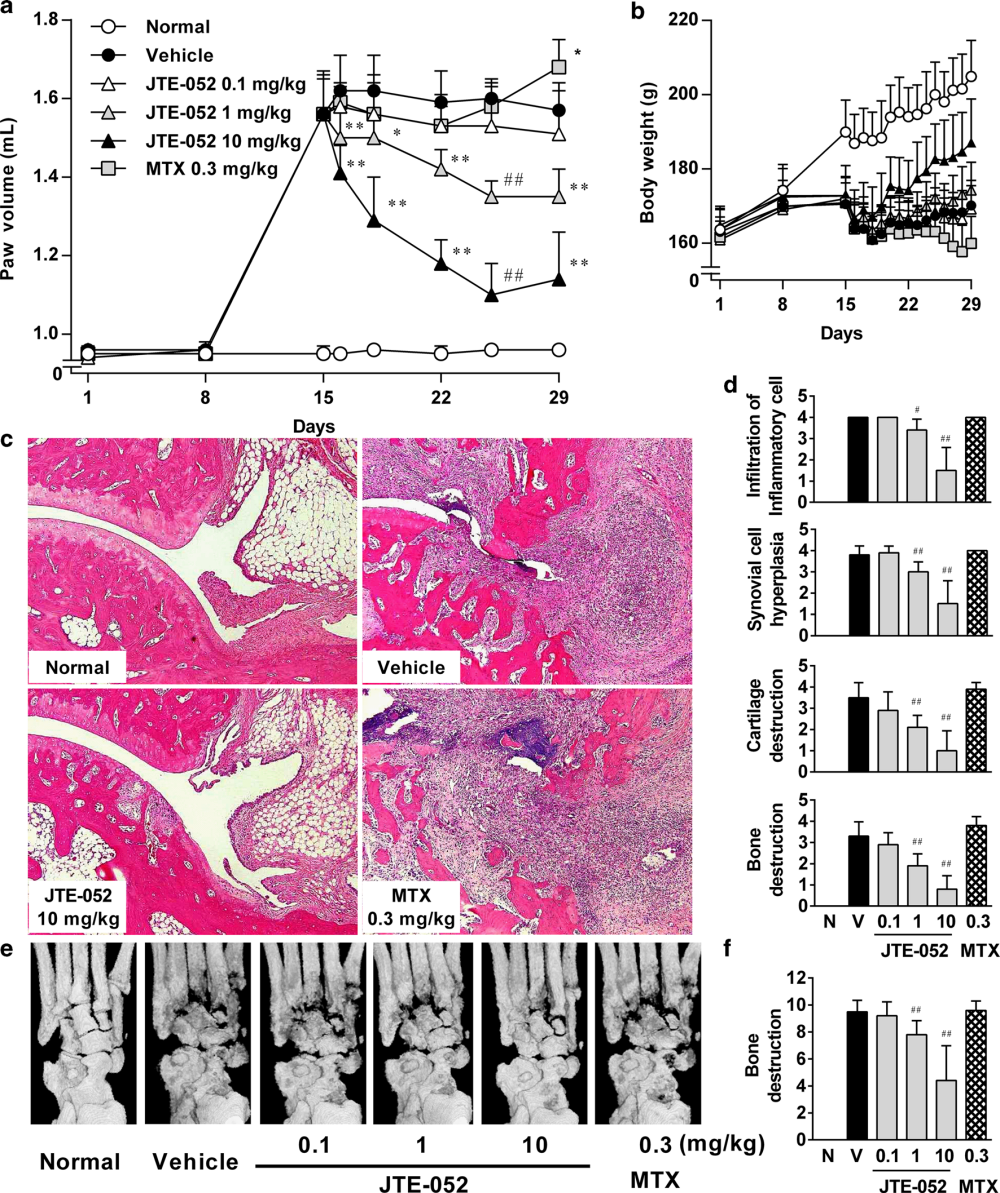
Therapeutic effects of JTE-052 on collagen-induced arthritis in a rat model. Rats with clinical signs of joint inflammation were randomized (*n* = 10) and orally administered vehicle, JTE-052 (0.1, 1, or 10 mg/kg), or MTX (0.3 mg/kg) once daily for 2 weeks from day 15. The hind paw volume (**a**) and body weight (**b**) were measured periodically. Images of hematoxylin and eosin staining (**c**) and radiographs of hindlimbs (**e**) are shown. Histological change (**d**) and radiographical change (**f**) were scored. The results are expressed as mean ± SD. *p < 0.05, **p < 0.01, versus vehicle by Dunnett’s test. ^#^ *p* < 0.05, ^##^ *p* < 0.01, versus vehicle by the Steel test

The effects of JTE-052 on arthritis development were investigated using a rat collagen-induced arthritis model, which is widely used as a disease model of RA. Collagen-induced arthritis was induced by injection of bovine type II collagen, and JTE-052 was administered before (from day 1) or after (from day 15) the onset of arthritis. In the administration from day 1, JTE-052 prevented the development of hind paw swelling and histological changes of inflammatory cell infiltration and synovial cell hyperplasia (Fig. 5a, c, d). JTE-052 inhibited radiographic and histological changes of bone destruction and cartilage destruction (Fig. 5c–f). These findings indicate protective effects of JTE-052 for joint inflammation and joint destruction. MTX also significantly inhibited these symptoms by administration from day 1 (Fig. 5a, c–f). In the administration from day 15, JTE-052 decreased the paw swelling in a dose-dependent manner (Fig. 6a). Improvement of paw swelling was noted on the day following initiation of the dosing. In addition, JTE-052 ameliorated the inflammatory cell infiltration, synovial cell hyperplasia, and cartilage/bone destructions in the histological and radiographic examinations at the end of the study (Fig. 6c–f). MTX did not attenuate all of these symptoms following its administration from day 15 (Fig. 6a, c–f).

## Discussion

In this study, we characterized the pharmacological profile of a novel JAK inhibitor, JTE-052. JTE-052 is highly specific for JAK family kinases and has a similar cytokine signaling inhibitory profile to tofacitinib. JTE-052 can inhibit the activity of various types of inflammatory cells and is efficacious even in MTX-resistant condition in rat arthritis model. In addition, JTE-052 demonstrated more potent effects on cytokine production than tofacitinib when the compounds were administered orally in the mouse IL-2-induced IFN-γ production model.

In the IL-2-induced IFN-γ production model in mice, oral dosing of JTE-052 and tofacitinib showed the time-dependent inhibitory effects, and the potency of JTE-052 was higher than that of tofacitinib at all time points examined. These results indicate that the in vivo potency of JTE-052 to suppress cytokine signaling was higher than that of tofacitinib in mice. Since JTE-052 had almost the same profile for inhibition of cytokine signaling as tofacitinib in the in vitro cellular experiments, JTE-052 might have better oral bioavailability or longer target residence time than tofacitinib in mice. Further examinations are required to explain the difference between the in vivo potency of the two compounds. Generally, it is postulated that the risk of off-target adverse events, such as liver injury, increases with higher amounts of drugs [19]. Given that the lower dosage of JTE-052 exhibited similar efficacy to the higher dosage of tofacitinib, JTE-052 may have an advantage over tofacitinib in the risk of off-target adverse events, such as transaminase elevation. However, this needs to be examined in a clinical study.

In the present study, we demonstrated differences in the pharmacological properties of the JAK inhibitors JTE-052, tofacitinib, and ruxolitinib. First, we examined the kinase inhibitory properties in kinase assays. JTE-052 showed a pan-JAK inhibitory profile by inhibiting all of the JAK activities in enzyme assays, and its inhibition of JAK1 and JAK2 was superior to that of JAK3 and Tyk2. Tofacitinib inhibited JAK1, JAK2, and JAK3 with nanomolar potency and Tyk2 with 10-fold weaker potency, consistent with a previous report [10]. These data indicate that JTE-052 has almost the same JAK inhibitory profile as tofacitinib, except for JAK3 inhibition, in which its potency was 7-fold weaker than that of tofacitinib. Ruxolitinib has been reported to inhibit JAK1 and JAK2 with nanomolar potency and JAK3 with 100-fold weaker potency [20]. However, in our results, ruxolitinib was more potent for JAK2 than for JAK1, and had an inhibitory effect on JAK3 with nanomolar potency. The difference between the previous report and our results for the potency of JAK inhibition by ruxolitinib may arise through the high ATP concentration in their assay system. The IC_50_ values in the previous report were determined at an ATP concentration of 1 mM, while the Ki values, which are not affected by the ATP concentration, were determined in our study. The data indicate that JTE-052 has almost the same JAK inhibitory profile as ruxolitinib, except for JAK2 and Tyk2 inhibition, in which these potencies were 5- to 8-fold weaker than those of ruxolitinib.

Next, we evaluated the inhibitory effects on cytokine signaling using IL-2, IL-6, IL-23, GM-CSF, and IFN-α. As JAK family kinases transduce cytokine signals into the nucleus with homodimeric or heterodimeric combinations of JAKs (JAK1/JAK3, JAK1/JAK2, JAK 2/Tyk2, JAK2/JAK2, or JAK1/Tyk2), we selected representative cytokine stimulations for the different combinations, i.e., IL-2 for JAK1/JAK3, IL-6 for JAK1/JAK2, IL-23 for JAK2/Tyk2, GM-CSF for JAK2/JAK2, and IFN-α for JAK1/Tyk2 (Table 3). JTE-052 and tofacitinib inhibited all of the signaling pathways induced by the cytokines we examined, but their potencies for the IL-23 and GM-CSF signaling pathways were weaker than for the other pathways. These findings are consistent with a previous report describing that tofacitinib has JAK1/JAK3 selectivity over JAK2 in cellular assays despite its potent JAK2 enzyme inhibition [10]. Interestingly, JTE-052 inhibited all of the examined cytokine signaling pathways with almost the same profile as tofacitinib, despite being less potent than tofacitinib for JAK3 enzyme inhibition. One potential explanation for this pattern is that JTE-052 has sufficient potential for JAK1 inhibition to inhibit the signaling at the receptors conjugated with JAK1 and JAK3. On the other hand, although ruxolitinib also inhibited all of the cytokine signaling pathways, the inhibitory potencies for IL-23, GM-CSF, and IFN-α were stronger than those of JTE-052 and tofacitinib. These results are consistent with the high potency of ruxolitinib for inhibition of JAK2 and Tyk2 compared with the other JAK inhibitors, since JAK2 or Tyk2 participates in these cytokine signaling pathways. It is thought that JAK2 is involved in erythropoietin signaling and important for erythrocyte development. Compared to ruxolitinib, JTE-052 might be expected to have a lower risk of anemia in the clinical setting owing to its relatively weak inhibition of JAK2/Tyk2 signaling pathway.

In the collagen-induced arthritis model, JTE-052 ameliorated the arthritic symptoms even when administered after the onset of arthritis. Under the same conditions, MTX failed to show inhibitory effects at the dose for which complete inhibitory effects were seen for its administration before the onset of arthritis. Currently, several pharmacological mechanisms for the action of MTX have been proposed, and reduction of antigen-dependent T cell proliferation is largely responsible for its anti-inflammatory effects [21]. It was also reported that tacrolimus, a calcineurin inhibitor, inhibited T cell proliferation but had no inhibitory effects in a rat collagen-induced arthritis model following administration after the onset of arthritis [15]. These findings indicate that anti-arthritic drugs that inhibit T cell functions are not able to ameliorate collagen-induced arthritis with administration after the disease onset. On the other hand, JTE-052 inhibited the functions of B cells, mast cells, and monocytes as well as those of T cells, indicating that JTE-052 is efficacious even in a therapeutic dosing regimen. From these findings, it is suggested that JAK inhibitors have additional pharmacological mechanisms compared with conventional DMARDs or immunosuppressants. These may be a reason why JAK inhibitors such as tofacitinib showed apparent effects in RA patients who had inadequate responses to MTX and in patients with various inflammatory diseases such as psoriasis, IBD, and transplantation rejection.

In conclusion, our findings demonstrate that JTE-052 is a novel JAK inhibitor with higher in vivo potency by oral administration and expected to ameliorate arthritis symptoms with lower dosing than tofacitinib. In addition, our findings demonstrate that JTE-052 has a distinct cytokine inhibitory profile from those of other JAK inhibitors. JTE-052 is expected to be a novel anti-inflammatory agent as a second generation JAK inhibitor for treatment of patients with autoimmune disease.

## Electronic supplementary material

Below is the link to the electronic supplementary material.

Supplementary material 1 (PDF 140 kb)
