# Supplementary material for: Pharmacological properties of JTE-052: a novel potent JAK inhibitor that suppresses various inflammatory responses in vitro and in vivo
Source: Inflamm Res. 2014 Nov 12;64(1):41–51. doi: 10.1007/s00011-014-0782-9 (PMC4286029; doi:10.1007/s00011-014-0782-9)

Pharmacological properties of JTE-052: a novel potent JAK inhibitor that suppresses various inflammatory responses in vitro and in vivo

Atsuo Tanimoto, Yoshihiro Ogawa, Chika Oki, Yukari Kimoto, Keisuke Nozawa, Wataru Amano, Satoru Noji, Makoto Shiozaki, Akira Matsuo, Yuichi Shinozaki, Mutsuyoshi Matsushita

Central Pharmaceutical Research Institute, Japan Tobacco Inc., 1-1 Murasaki-cho, Takatsuki, Osaka 569-1125, Japan

Supplement

Table S1 pKi values of JAK inhibitors in enzyme assays

Compounds		JAK1	JAK2		JAK3		Tyk2	
JTE-052	pKi	8.7 ± 0.1	8.8 ± 0.0		8.3 ± 0.0		7.9 ± 0.1	
Tofacitinib	pKi	8.7 ± 0.1	8.8 ± 0.1		9.1 ± 0.0	‡	7.7 ± 0.1	
Ruxolitinib	pKi	8.9 ± 0.2	9.5 ± 0.3	‡	8.2 ± 0.2		8.8 ± 0.1	‡

Values are means ± SEM. Three independent experiments were performed.
‡ $p < 0.01$ versus JTE-052 by Dunnett's test.

Fig. S1 Inhibitory effects of JTE-052 on the activation of various inflammatory cells. (a) Human T cells, (b) human B cells, (c) human monocytes, and (d) human mast cells were stimulated with cytokines and their proliferations and inflammatory cytokine productions were measured as described for Figure 3. Three independent experiments using cells from different donors were performed, and the two experiments other than the representative data in Figure 3 are shown. Values are the means of 3–6 samples for each experiment.

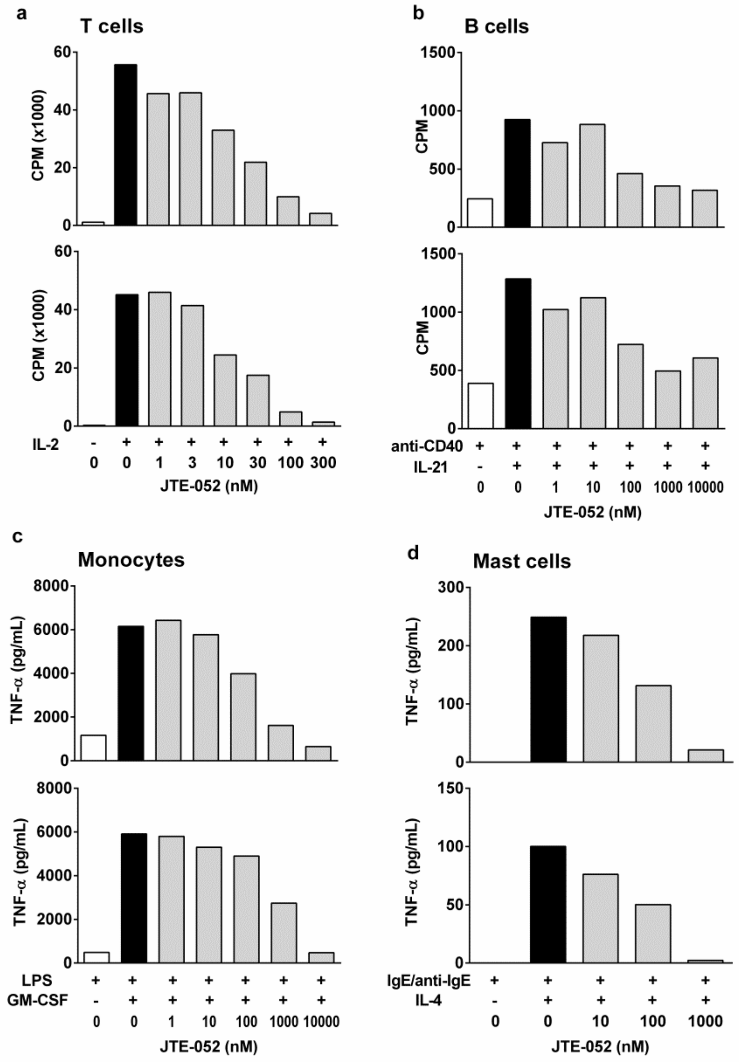

Supplement: Supplementary file 1 — Supplementary material 1 (PDF 140 kb) [file 11_2014_782_MOESM1_ESM.pdf]
